# Prophylaxis and Treatment of *Pneumocystis jiroveci* Pneumonia in Lymphoma Patients Subjected to Rituximab-Contained Therapy: A Systemic Review and Meta-Analysis

**DOI:** 10.1371/journal.pone.0122171

**Published:** 2015-04-24

**Authors:** Xuqin Jiang, Xiaodong Mei, Di Feng, Xiaojing Wang

**Affiliations:** Department of Respiratory Medicine, Anhui Provincial Hospital, Hefei, China; Albert Einstein College of Medicine, UNITED STATES

## Abstract

*Pneumocystis jiroveci* pneumonia (PCP) is frequently reported in lymphoma patients treated with rituximab-contained regimens. There is a trend toward a difference in PCP risk between bi- and tri-weekly regimens. The aims of this systemic review and meta-analysis were to estimate the risk for PCP in these patients, compare the impact of different regimens on the risk, and evaluate the efficacy of prophylaxis. The cohort studies with incept up to January 2014 were retrieved from the Cochrane Library, Medline, Embase, and Web of Science databases. Studies that compared the incidence of PCP in patients with and without rituximab treatment were conducted. Studies that reported the results of prophylaxis were concentrated to evaluate the efficacy of prophylaxis. Fixed effect Mantel-Haenszel model was chosen as the main analysis method. Funnel plots were examined to estimate the potential selection bias. Egger’s test and Begg’s test were used for the determination of possible small study bias. Eleven cohort studies that met the inclusion criteria were finally included. Results indicated that rituximab was associated with a significantly increased risk for PCP (28/942 vs 5/977; risk ratio: 3.65; 95% confidence interval 1.65 to 8.07; *P*=0.001), and no heterogeneity existed between different studies (*I^2^*=0%). Little significant difference in PCP risk was found between bi-weekly and tri-weekly regimens (risk ratio: 3.11; 95% confidence interval 0.92 to 10.52, *P*=0.068). PCP risk was inversely associated with prophylaxis in patients treated with rituximab (0/222 vs 26/986; risk ratio: 0.28; 95% confidence interval 0.09 to 0.94; *P*=0.039). In conclusion, PCP risk was increased significantly in lymphoma patients subjected to rituximab-contained chemotherapies. Difference in PCP risk between bi-weekly and tri-weekly regimens was not significant. Additionally, prophylaxis was dramatically effective in preventing PCP in rituximab-received lymphoma patients, suggesting that rituximab should be recommended for these patients.

## Introduction

*Pneumocystis jiroveci* pneumonia (PCP) is an opportunistic infection which occurs in immunosuppressed patients such as those infected with the human immunodeficiency virus (HIV) [1]. In recent years, PCP has also been frequently reported in lymphoma patients treated with rituximab-contained regimens [2–4], and the increase of PCP in these patients was considered to be related to rituximab. Rituximab is a chimeric monoclonal antibody, which targets B cell-specific antigen CD20. It can reduce the number of B cells and remarkably enhance the efficacy of chemotherapy in non-Hodgkin lymphoma patients. Therefore, rituximab has been recommended as a first-line therapy for non-Hodgkin lymphoma since 2006 [5]. Along with the widespread application of rituximab, the incidence of PCP also increases rapidly [2–4]. Many studies show that the risk for PCP in patients with lymphoma increases with rituximab therapy [6–8]. The reported incidence rate in these patients could be as high as 10.04 to 13.04% [9,10]. Meanwhile, other studies claimed that rituximab was not a risk factor for PCP [11]. In contrast to recent reports, no PCP case was reported in a large-scale clinical trial of rituximab (n = 3,000) [12–16].

The clinical course of PCP in lymphoma patients subjected to rituximab can be quite fulminant with high mortality, which has been reported as high as 33.3% [17]. Sudden deaths have been reported in some patients given with anti-pneumocystis treatments [17,18]. In view of the increased incidence and potential fatality of PCP, the role of prophylaxis has been studied [19–22]. Prophylaxis was found to be highly efficient in preventing PCP [4,19,20] without the serious side effects of other anti-pneumocystis drugs [20]. Therefore, prophylaxis is strongly recommended for patients receiving therapies with rituximab [4,19,20]. Some researchers have argued against the use of universal prophylaxis since the incidence of PCP was not remarkably high and the use of the anti-pneumocystis drug, trimethoprim-suffamethoxazole (TMP/SMZ) might cause bone marrow suppression [22].

Therefore, it is still unclear whether prophylaxis should be recommended in lymphoma patients subjected to rituximab. There is also a need to study the risk of PCP associated with rituximab treatment and the exact incidence rate of PCP. Herein, we performed a systemic review and meta-analysis on clinical trial data to address these issues.

## Methods

In Mar 2013, we reported two cases of patient with non-Hodgkin disease who developed PCP during rituximab-contained chemotherapy and reviewed related literature concentrating on the incidence of PCP in these patients [3]. We found that the incidence of PCP and the use of prophylaxis remained controversial. We also failed to find any randomized controlled trials for this query. Further discussion of the review design and protocol took place during the third quarter of 2013, based on the meta-analytical techniques to evaluate the correlation between rituximab and PCP in lymphoma patients. Final consensus on the protocol was reached at the end of December 2013, and the performance of this review began in January 2014. Meta-analyses of observational studies were performed following the standard criteria [23]. The study was approved by the independent ethics committee (IEC) of Anhui Provincial Hospital (No2013045).

### Literature Search

We searched the Cochrane library, Medline, Web of Science, and Embase electronic databases from inception to January 2014 for relevant articles. Since PCP was an infrequent complication, which may not be the main purpose of the studies, a broad eligibility strategy was used to capture all potentially relevant data by using the following search string: “rituximab” and “pneumocystis pneumonia”. References of included studies were manually inspected for more trials. Standard guidelines for conducting and reporting meta-analyses of observational studies were followed. No language or publication restrictions were applied.

### Study selection

Two reviewers independently screened the titles and abstracts of all possible relevant articles after removal of duplicates. Then, articles that were clearly not relevant-such as editorials, reviews, and single case study reports were removed. Potentially relevant articles were obtained in full text and read independently by two reviewers to determine the eligibility for inclusion. Trial inclusion criteria mandated the incidence of pneumocystis infection as an outcome. Only cohort studies that presented the numbers of PCP case in lymphoma patients treated with and without rituximab were included. For analyses of the effectiveness of prophylaxis, cohort studies that reported the results of prophylaxis were concentrated. As the incidence rate of PCP in lymphoma patients treated with rituximab was the focus of this review and there was immunological abnormality in patients with HIV infection, we excluded studies if, in which, the patient had concomitant HIV infection. We obtained copies of all articles identified as being potentially relevant, including contacting authors as necessary.

### Data extraction

Two reviewers independently extracted the relevant characteristics and outcomes from eligible trials. Extracted data were directly imported to Microsoft excel sheets, which included predefined fields set up to capture aspects of study design and quality as well as all results (PCP case and total patient numbers, chemotherapy regimens, chemotherapy cycles). We also extracted additional data about age, sex, diagnostic methods, white blood cell count numbers, the onset time of PCP after rituximab treatment, pharmaceuticals used for prophylaxis, and outcome of treatment. When necessary, we contacted the corresponding authors for details on specific aspects of the data. For those that did not give the numbers of PCP cases and total patients directly, we calculated that from the original tables or article contents. Methodological quality of studies was evaluated using the Newcastle-Ottawa scale (NOS) for assessing non-randomized studies used in meta-analyses [24], as suggested by the Cochrane handbook of systematic reviews and meta-analyses [25]. Disagreements were resolved after rechecking the source articles and further discussion among the reviewers.

### Statistical analysis

Software Stata 11.0 (Stata Corporation, College Station, TX) was used to conduct meta-analysis. Risk ratio (RR) with 95% confidence interval was calculated. Heterogeneity between studies was tested using the *I* ^2^ statistic [26,27]. Fixed effect Mantel-Haenszel model was chosen as the main analysis method when the heterogeneities were confirmed not statistically significant. Otherwise, random-effect model would be used and sensitivity analysis performed. Funnel plots were examined visually to estimate potential selection bias (publication or other factors). Egger’s test and Begg’s test were used for determine possible small study bias. Peto odds ratio (OR) analysis was also conducted and compared with Mantel-Haenszel model, because it can provide the best confidence interval coverage and be more powerful in dealing with low event rates in such circumstances compared with the validity of fixed effect assumption [28]. All the *P* values were two-sided, and statistical significance was defined at the 0.05 level.

## Results

### Literature search

**Fig 1:**
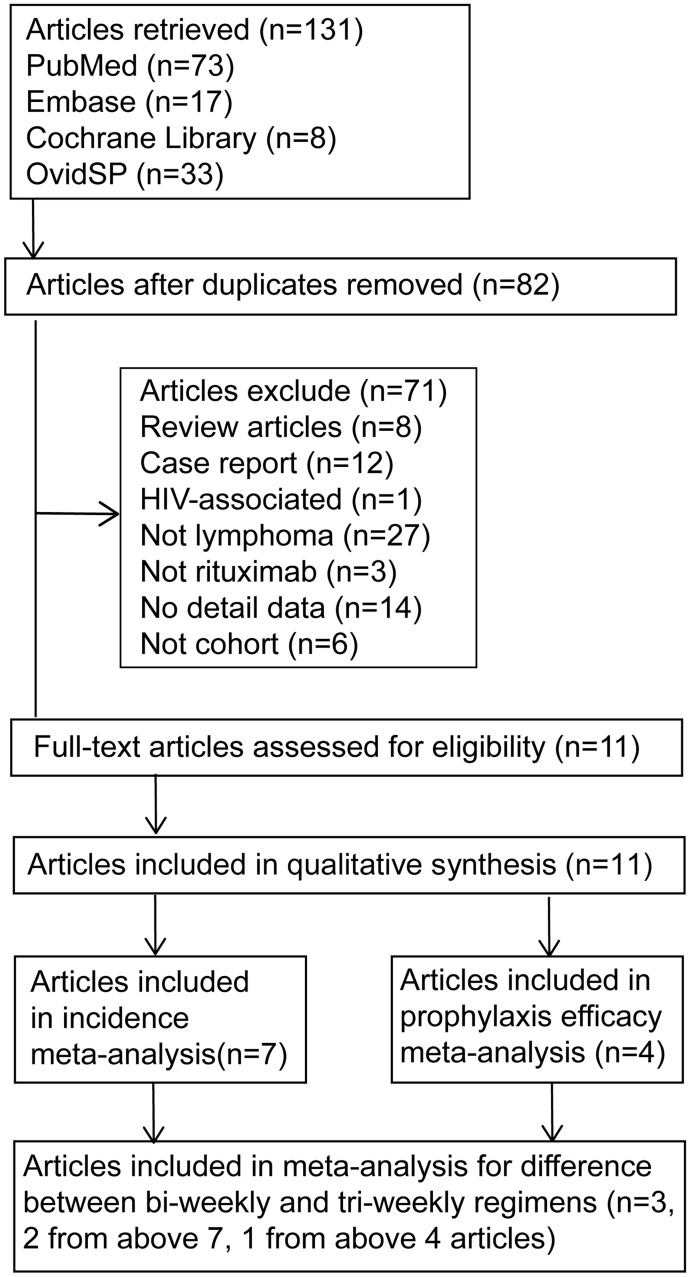
Flow diagram of identification process for eligible studies.

The flow diagram of article selection is shown in Fig 1. Initially, 131 articles were found. After removal of the duplicates, 82 articles were identified. Further, 71 studies were excluded based on our exclusion criteria. Of those excluded studies, there were eight reviews [29–36], six studies recruited patients with nephrotic syndrome [37,38], renal transplantation [37–42], three with other solid organ transplantation [43–45], six with systemic lupus erythematosus [46–51], seven with chronic lymphocytic leukemia [52–58], two with antibody autoimmune haemolytic anemia [59,60], two with macroglobulinemia [61,62], and one with Wegener’s granulomatosis [63]. Three studies in which rituximab was not used [64–66], 12 case reports [2–4,38,67–74], and six non cohort trials were also excluded [9,18,75–78]. Eleven studies were included for further investigation. Eventually, seven cohort studies that met the inclusion criteria were chosen for meta-analysis to compare PCP risk in patients with and without rituximab. One of the seven studies contained four groups of patients treated with different chemotherapy regimens and intervals. We treated this study as four trials. For one of the four trials presented no PCP case in neither of the two groups, it was excluded in meta-analysis [10]. Eventually, nine trials were included for meta-analysis. Three trials [10,17,20] that represented the occurrence of PCP in patients received R-CHOP tri-weekly (R-CHOP-21) and bi-weekly (R-CHOP-14) were included to assess the affect of different chemo-regimens and intervals on PCP risk. In addition, four articles [19–22] containing five trials that presented the results of prophylaxis were included for meta-analysis to evaluate the efficacy of prophylaxis.

### Study characteristics

Seven cohort studies included 1919 participants, of whom 942 were treated with rituximab and 977 treated without rituximab. The characteristics of these trials are shown in Table 1. Six of the seven studies were from Asia. The other one was from Norway, in which, the authors reported four groups of patients treated with different regimens [10]. Six trials enrolled patients with non-Hodgkin lymphoma of miscellaneous types [6,7,10,11,17,79], while one study enrolled patients with diffuse large B cell lymphoma merely [80]. The cohort size of studies ranged from 100 to 529 cases. In diagnostic methods for PCP, PCR was used in three studies [6,7,10], microscopy examination in two studies [11,80], β-glucan detection in two studies [6,17], and direct fluorescentantibody assay used in one study [79]. Rituximab-added chemotherapy tri-weekly was administered in four studies and bi-weekly in two others. One study which included both bi-weekly and tri-weekly regimens was treated as two trials in meta-analysis [10]. In meta-analysis to assess the difference between bi-weekly and tri-weekly rituximab-added regimens in PCP incidence, three studies [10,17,20] were concluded. One of the studies that included CHOP and cyclophosphamide, doxorubicin, vincristine, etoposide, and prednisolone (CHOEP) regimens was treated as two trials, Table 2. Four articles containing five trials were included in meta-analysis to determine the efficacy of prophylaxis [19–22]. The characteristics of these studies were shown in Table 3.

**Table 1:** Characteristics of cohort studies included in PCP risk meta-analysis for patients with and without rituximab.

Author(year)	Country	Disease	Cohort size	Diagnostic method	Chemo-regimen	Chemo-cycle (day)	Onset time of PCP	Outcome	
								Cured	died
Ennishi (2008)	Japan	NHL	195	PCR	(R-)CHOP	NG	60–120d	2	0
Huang (2011)	China	DLBCL	529	microscopy	(R-)CHOP	21	4(1–7)cycle	NG	
Kato (2011)	Japan	NHL	103	microscopy	(R-)CHOP	NG	212d	1	0
Katsuya (2009)	Japan	NHL	188	PCR/β-glucan	(R-)CHOP	21/14	4,6,7cycle	2	1
Kolstad a (2007)	Norway	NHL	71	PCR	(R-)CHOEP	14	NG	6	0
Kolstad b (2007)	Norway	NHL	417	PCR	(R-)CHOP	21	NG	2	0
Kolstad c (2007)	Norway	NHL	81	PCR	(R-)CHOP	14	NG	2	0
Kurokawa (2010)	Japan	NHL	235	PCR/β-glucan	(R-)CHOP	NG	2,3,4,4,5cycle	4	1
Lim (2010)	Korea	NHL	100	DFA	(R-)CHOP	21	27–117d	NG	

DFA: direct fluorescent antibody assay; DLBCL: diffuse large B cell lymphoma; ND: not do; NG: not given; NHL: non-Hodgkin’s lymphoma; NOS: Newcastle-Ottawa quality assessment scale; PCR: polymerase chain reaction; R-CHOP: rituximab plus cyclophosphamide, doxorubicin, vincristine, and prednisone; R-CHOEP: rituximab plus cyclophosphamide, doxorubicin, vincristine, prednisone and etoposide

**Table 2:** Characteristics of cohort studies reporting PCP occurrence in bi-weekly and tri-weekly rituximab-contained therapies.

Author (year)	Country	Disease category	Cohort size	Diagnostic method	Regimens	No of pts in therapy		Onset time of PCP	Outcome		
						bi-weekly	tri-weekly		Cured	det	died
Katsuya (2009)	Japan	NHL	129	PCR/β-glucan	R-CHOP	2	127	4,6,7cycle	2	0	1
Kolstad a (2007)	Norway	NHL	55	PCR	R-CHOEP	46	9	NG	6	0	0
Kolstad b (2007)	Norway	NHL	46	PCR	R-CHOP	32	14	NG	4	0	0
Hardak (2012)	Israel	DLBCL	132	PCR	R-CHOP	85	47	2,5,5,6,6cycle	0	3	2

det: deteriorated; DLBCL: diffuse large B cell lymphoma; NHL: non-Hodgkin’s lymphoma; NG: not given; NOS: Newcastle-Ottawa quality assessment scale; pts: patients. PCR: polymerase chain reaction; R-CHO(E)P: rituximab plus cyclophosphamide, doxorubicin, vincristine, (etoposide), prednisone

**Table 3:** Characteristics of studies included for meta-analysis to determine the efficacy of prophylaxis.

Author (year)	Country	Disease	Cohort size	Diagnostic method	Regimens	Chemo-cycle (day)	Onset time of PCP	Outcome		
								Cured	det	died
Haeusler (2013)	Australia	NHL	66	PCR	FCR	28	3–6cycle	1	0	0
Hardak a (2012)	Israel	DLBCL	85	PCR	R-CHOP	14	2,5,5,6,6cycle	NG		
Hardak b (2012)	Israel	DLBCL	47	PCR	R-CHOP	21	2,5,5,6,6cycle	NG		
Hashimoto (2010)	Japan	NHL	297	PCR/CT/β-glucan	R-CHOP	14	2,2,4,4,5,5cycle	NG		
Kim (2012)	Korea	NHL	713	IF+imageology	R-CHOP	21	NG	1	12	1

CT: computed tomography; det: deteriorated; DLBCL: diffuse large B cell lymphoma; FCR: fludarabine, cyclophosphamide and rituximab; IF: immunofluorescence, NG: not given; NHL: non-Hodgkin’s lymphoma; NOS: Newcastle-Ottawa quality assessment scale; PCR: polymerase chain reaction; R-CHOP: rituximab plus cyclophosphamide, doxorubicin, vincristine, and prednisone

### Study quality

The quality of trials included for meta-analysis was satisfactory and most trials were judged to be at low risk of bias. Out of seven studies, five studies had medium to high NOS scores, and the other two had medium to low scores: one article did not give the information of grouping method [80] and the other lacked sufficient information to determine an overall score (presented the results table only) [10]. Removal of studies with medium to low NOS scores did not greatly change the summary estimate. Two studies in meta-analysis for assessing the difference between bi-weekly and tri-weekly chemotherapies were scaled as high quality [17,20] while the other study could not be scaled [10]. Four studies [19–22] including five trials for meta-analysis to evaluate the efficacy of prophylaxis had high quality NOS scores too.

### Incidence rate of PCP

**Fig 2:**
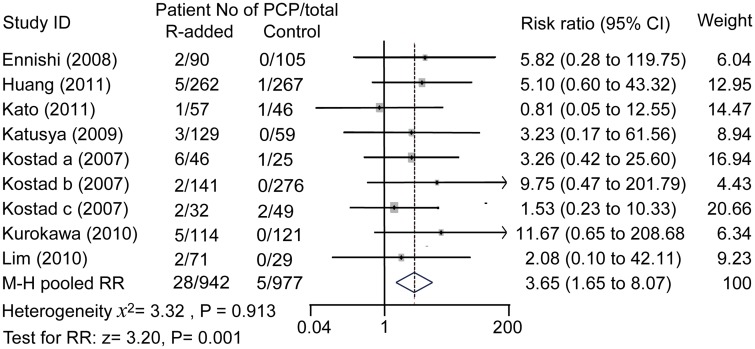
Effect of rituximab treatment on PCP risk. M-H pooled risk ratio = 3.65, fixed effect model method. R: rituximab. Rituximab increased the risk for PCP in lymphoma patients significantly.

Results showed that the incidences of PCP in lymphoma patients with rituximab-contained regimens were higher than that in those without rituximab-contained regimens (28/942 vs 5/977). Forest figure results showed that patients subjected to rituximab had a higher risk of PCP (RR: 3.65, 95% confidence interval: 1.65 to 8.07; *P* = 0.001), and there was no heterogeneity between studies (*I* ^2^ = 0%, *χ* ^2^ = 3.32; *P* = 0.913, Fig 2).

### Difference between bi-weekly and tri-weekly regimens

**Fig 3:**
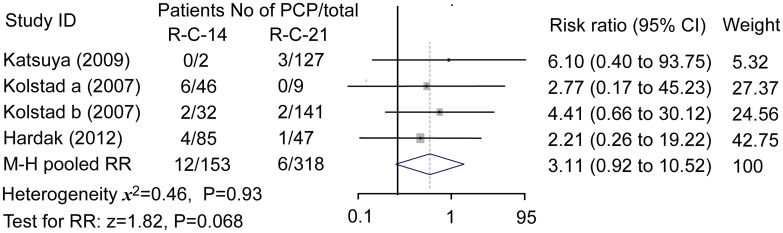
PCP risk in bi-weekly and tri-weekly regimens. M-H pooled risk ratio = 3.11; fixed effect model method. R-C-14: rituximab-added chemotherapy bi-weekly; R-C-21: rituximab-added chemotherapy tri-weekly. Patients treated with bi-weekly regimen seemed to have a higher risk for PCP but the difference between the two regimens was not statistically significant.

Data of three studies containing four trials were meta-analyzed. The incidence of PCP in patients treated with bi-weekly regimen seemed higher than that with tri-weekly regimen (12/165 vs 6/324). Fixed effect model showed M-H pooled RR was 3.11 (95% confidence interval 0.92 to 10.52, *P* = 0.068), which did not indicated a statistically significant difference between the two regimens although a tendency seemed existed, Fig 3.

### Effectiveness of prophylaxis

**Fig 4:**
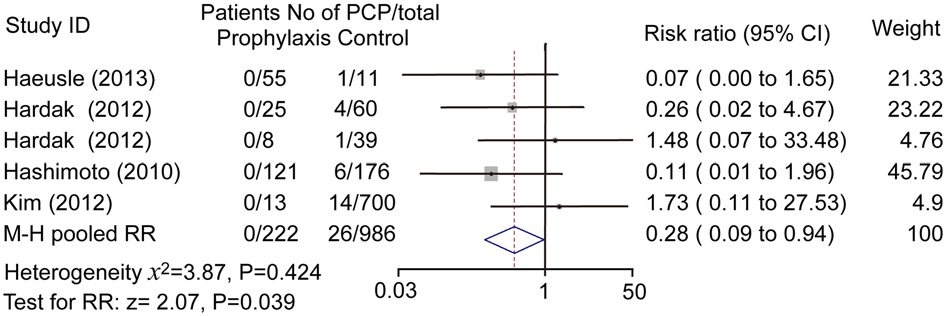
Effect of prophylaxis on PCP risk in rituximab-received lymphoma patients. M-H pooled risk ratio = 0.28, fixed effect model method. Prophylaxis dramatically reduced PCP risk in rituximab-received patients.

Prophylaxis was effective as there are remarkable differences between patients with and without prophylaxis (0/222 vs 26/986). Pooled estimate demonstrated a statistically difference in the two groups (RR: 0.28; 95% confidence interval 0.09 to 0.94; *P* = 0.039), Fig 4. Pooled odds ratio was 0.17 (95% confidence interval 0.05 to 0.55, *P* = 0.003), which was consistent to the result from Mantel-Haenszel fixed effect model.

### Heterogeneity and sensitivity

Evidence of statistical heterogeneity was lacking between the included cohort studies. Heterogeneity chi-square = 3.32, *P* = 0.913. I-squared (variation in RR attributable to heterogeneity) = 0%. Sensitivity analysis indicated that omission of any studies did not affect the pooled estimate significantly. No heterogeneity was found between bi-weekly and tri-weekly regimens (Heterogeneity chi-squared = 0.46, *P* = 0.927; I-squared = 0.0%), or received prophylaxis or not (Heterogeneity chi-squared = 3.87; *P* = 0.424; I-squared = 0.0%). We did not perform sensitivity analysis in the latter two meta-analyses due to their limited number of included trials.

### Publication bias

**Fig 5:**
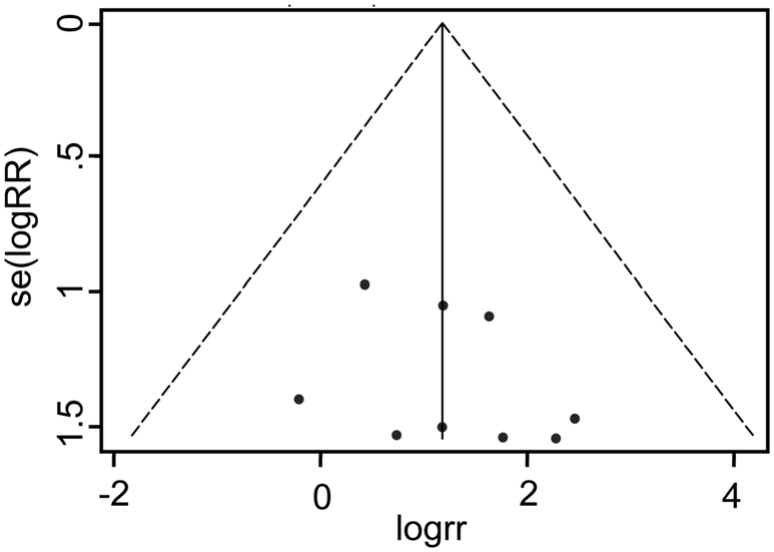
Funnel plot (with pseudo 95% confidence limits) for rituximab on PCP risk.

Funnel plot did not show a significant publication bias among cohort studies that presented PCP incidence in patients with or without rituximab treatment, Fig 5. Furthermore, no indication of publication bias was found in Egger's test (*t* = 0.95, *P* = 0.347) and Begg's test(z = 0.94, *P* = 0.348, continuity corrected)either. Similarly, no significant difference was found among the studies included for meta-analysis of bi-weekly or tri-weekly therapy and prophylaxis.

## Discussion

This meta-analysis, based on data of cohort trials, demonstrated that rituximab was associated with a significant increase of PCP risk in lymphoma patients. Based on our analysis, no difference was found in PCP risk between different regimens; meanwhile, anti-pneumocystis prophylaxis was effective in these patients. There was no evidence of methodological heterogeneity or substantial evidence of publication bias.

The incidence of PCP in lymphoma patients subjected to rituximab treatment was a concern and warranted in daily practice. Rituximab can improve the efficacy of CHOP-like therapy by affecting B cells. However, as B cells play a vital role in generation of CD4+ T cells for defending *Pneumocystis jiroveci* infection in the lungs [81], reduction of B cells could lead to insufficient generation of CD4+ T cells and subsequently higher risk for *Pneumocystis jiroveci* infection. Rituximab has been reported to be associated with PCP in a chronic childhood autoimmune hemolytic anemia patient [60], and suspected to increase risk for PCP in lymphoma patients [2–4,8,68,69]. However, the correlation between PCP and rituximab and the exact incidence of PCP remain controversial. For example, one study claimed no correlation between rituximab and the increase of PCP [11], while others indicated a notable increase of PCP cases and presumed this increase was correlated with rituximab. Unsatisfactorily, many of these studies were case reports rather than randomized controlled trials or cohort trials. Therefore, the exact incidence of PCP remained under debate. In our meta-analysis of cohort trials, a significant increase of PCP risk was confirmed.

Many factors could affect the occurrence of PCP, such as administration of corticosteroids [59,79], shorter intervals of chemotherapy [10,17,20], and decrease of white blood cells [17,18] especially CD4+ lymphocytes [18,19,80]. Patients treated with bi-weekly regimen were reported to have a higher risk of PCP [20]. In our meta-analysis, a higher risk for PCP in patients treated with biweekly regimen was not established. However, bi-weekly and tri-weekly regimens are both routine therapies for lymphoma patients in daily practice. Due to the limited number of studies in our meta-analysis, further studies with large number of patients are needed to compare the difference between the two regimens.

There is also a need to investigate the role of prophylaxis since there are divergent views on whether anti-pneumocystis prophylaxis is necessary for patients subjected to rituximab-added chemotherapy. In view of the increased incidence and fatality of PCP, some researchers administered prophylaxis [9,10,20–22,76,78] and recommended it as a routine use [4,9,10,18,20]. It was demonstrated that with adequate prophylaxis, very few PCP occurred in those patients, and no adverse reactions from TMP-SMX were observed [6], suggesting the efficacy and safety of prophylaxis. Our meta-analysis also showed that pooled RR of PCP was reduced when prophylaxis was given. Similarly, a previous systematic review reported the efficacy of prophylaxis for PCP in immunocompromised non—HIV-infected patients [35]. Twelve randomized trials, which included 1245 patients who had undergone autologous bone marrow or solid organ transplant or had hematologic cancer, were included in the review. When TMP/SMZ was administered, the occurrence of PCP was reduced by 91%. These results were consistent with that of our meta-analysis although the patient populations were different.

### Strengths of the study

To our knowledge, this is the first meta-study that covers the available evidence and performs a quantitative estimate of the impact of rituximab on the risk for PCP in lymphoma patients. The strengths of this meta-analysis lay in the broad search from multiple online databases, covering published literature from inception to newly updated, the high sensitivity terms that we used to obtain literature, and further hand searching combined with checking reference lists of included studies. We also contacted authors when necessary and carefully calculated data from articles when the data were not provided directly. Heterogeneity between studies was not obvious which indicated consistency in these studies. Besides, we did not find considerable evidence of publication bias, although this cannot be completely excluded. Our meta-analysis is robust based on the analysis from the funnel plot, Egger’s test and Begg’s test.

In meta-analysis for effectiveness of prophylaxis, RR and OR values were both calculated and compared. In contrast to the Mantel-Haenszel model, Peto method was more sensitive in estimating the true strength of the association for an event of low occurrence rate. As PCP is an opportunistic infection and the incidence might be lowered when prophylaxis was administered, we generated Peto method with OR rather than fixed effect model with RR in this meta-analysis, to improve the quality of our analysis.

### Limitations of the review

Nevertheless, there were some limitations in our analysis due to the varied quality of retrieved articles. The included trials were all cohort studies, which are not as robust as randomized controlled trials. This might lead to some biases, particularly selection and attrition biases. In addition, there are differences in patient populations, sample size, number of patients in rituximab-added groups versus controls, chemotherapy cycles, intervals of therapy, length of follow up, and diagnostic methods for PCP among different studies. We could not analyze the importance of other possible factors in great depth either because of the lack of the details. The number of trials included to assess the difference between bi-weekly and tri-weekly regimens, and the effectiveness of prophylaxis was also limited. The estimates might not be precise owing to the fairly low event rate of PCP. To further assess PCP risk in different therapies such as bi-weekly or tri-weekly regimens and guide the duration of prophylaxis, further studies are needed.

## Conclusions

Our meta-analysis demonstrated that the risk for PCP was associated with rituximab. Patients with lymphoma subjected to rituximab-added therapy had an increased risk of PCP. No difference was observed between bi-weekly and tri-weekly chemotherapies in PCP risk based on the limited number of studies included in our analysis. Prophylaxis was very effective in preventing this life-threatening complication. Therefore, clinicians should be more vigilant in view of the increased risk of PCP and prophylaxis should be recommended as a routine practice.

## Supporting Information

S1 Table PRISMA Checklist. (DOC)
